# Long-term influences of pipe materials on bacterial communities of matured biofilms (> 40 years’ old) in drinking water distribution systems

**DOI:** 10.1016/j.fmre.2024.05.019

**Published:** 2024-06-29

**Authors:** Anran Ren, Jun Li, Zhen Zhang, Ed van der Mark, Lihua Chen, Xiaoming Li, Walter van der Meer, Gang Liu

**Affiliations:** a Key Laboratory of Drinking Water Science and Technology, Research Center for Eco-Environmental Sciences, Chinese Academy of Sciences, Beijing 100085, China; b University of Chinese Academy of Sciences, Beijing 100049, China; c General Office, the People's Government of Xinglong prefecture, Chengde 067300, China; d Dunea Water Company, Zoetermeer P.O. Box 756, the Netherlands; e Sanitary engineering, Department of Water management, Faculty of Civil Engineering and Geosciences, Delft University of Technology, Delft P.O. Box 5048, the Netherlands; f Science and Technology, University of Twente, Enschede P.O. Box 217, the Netherlands; g Oasen Water Company, Gouda P.O. Box 122, the Netherlands

**Keywords:** Pipe material, Biofilm communities, Environmental selection, Harmonizing effects, Long-term influences

## Abstract

Pipe materials appear to play an important role in the development of biofilms in drinking water distribution systems. However, there is controversy as to whether pipe materials shape the composition and diversity of bacterial communities in biofilms. To investigate the long-term effects of pipe materials on biofilms, triplicate samples of mature biofilms on unplasticized polyvinyl chloride (PVC-U), grey cast iron and asbestos cement (pipe age > 40 years) were obtained from three areas of an unchlorinated drinking water distribution system in the Netherlands. Illumina sequencing was performed and 773 OTUs (730 OTUs-814 OTUs) were detected within the biofilms on the three pipe materials, all of which were dominated by Proteobacteria (36.2%-46.1%). Both the alpha and beta diversity results showed that the bacterial communities of the biofilms formed on different pipe materials were highly similar. The neutral community model revealed that the assembly of the biofilm communities was governed by environmental selection rather than neutral processes. Among the 142 shared OTUs between the water and biofilm samples, there were 25 enriched OTUs (e.g., OTU7, assigned as *Nitrospira* spp.), which accounted for 62.6% of the total sequences, while 16 OTUs were disadvantaged (e.g., OTU14 and OTU40, assigned as Hyphomicrobiaceae), accounting for 2.2% of the sequences. Based on the findings, we propose and discuss a harmonisation process by which biofilms with significant differences due to the pipe material harmonize over time resulting in biofilms with similar bacterial communities. Our findings provide valuable insights into long-term biofilm development, bridging an essential gap in our current understanding of the influence of pipe materials on biofilm communities. These findings also highlight the importance of long-term studies and point to a potentially masked harmonizing process during biofilm development over years/decades.

## Highlights

• Dominated by Proteobacteria, biofilm communities on different pipe material were similar.
• The 25 enriched OTUs, 17.6% of the shared OTUs, accounted for 62.6% of the sequences.
• Biofilm communities were strongly assembled by selection rather than neutral process.
• The significant contribution of pipe material might be harmonized over years/decades.

## Graphical abstract

**Figure:**
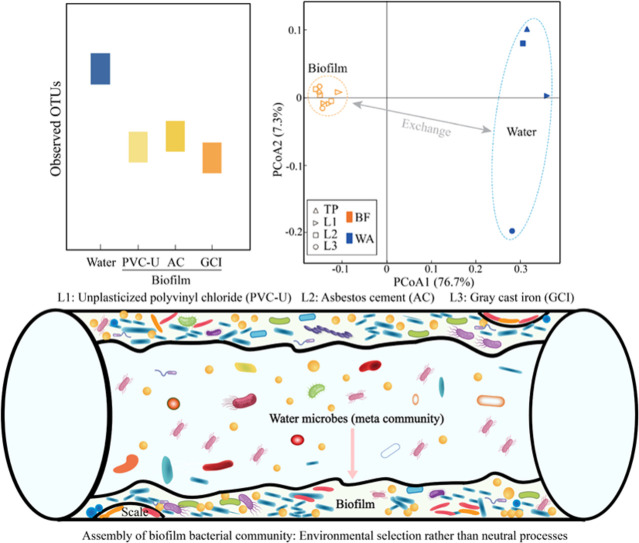

## 1 Introduction

Biofilms formed on the inner surface of drinking water distribution pipes are a complex mixture of microbes and organic and inorganic materials that have accumulated within a microbially-produced polymeric matrix [1,2]. Despite the maintenance of disinfectant residuals, the formation of biofilms is unavoidable and unwanted in drinking water distribution systems (DWDSs) [[3], [4], [5]] because biofilms are a reservoir for (opportunistic) pathogens [6], they may cause microbial corrosion [7], and they continuously release microbes into bulk water [8], especially during changes in the quality of supply water [9,10]. Therefore, biofilms have attracted increasing research attention over the last few decades. For example, studies have examined the biofilm formation potential of both pilot and full-scale distribution systems [4,11], the quantity and communities of biofilms [12,13], biofilm prevention and inhibition by nanomaterials [[14], [15], [16], [17]], the (opportunistic) pathogens in biofilms [6,18,19] and the key factors for the development and management of biofilms [[20], [21], [22], [23]].

Typically, the pipelines of DWDSs range in length from tens to several hundreds of kilometres, e.g., 0.4 million kilometres in the Netherlands [24] and 1.1 million kilometres in China, with 20,000 km of water pipelines in the city of Beijing alone [25]. The material that makes up the distribution pipes that are in contact with drinking water is important in terms of its potential contribution to water quality deterioration and energy consumption [26]. This is especially true when considering its significant influences on planktonic bacterial growth and biofilm formation [27,28]. However, there is controversy in the literature regarding how pipe materials can affect microbial communities of biofilms, with studies debating if the pipe material influences the composition and diversity of bacterial communities. Several researchers have found significant differences in the bacterial communities of biofilms formed on different pipe materials [[29], [30], [31], [32]], while others have found similar bacterial communities among biofilms formed on different pipe materials [[33], [34], [35]]. Though valuable knowledge has been obtained, the critical differences in the scale (pilot vs. full scale), duration (days vs. years) and sampling strategies (flushing vs. swabbing) of reported studies mean it is impossible to make reasonable cross-comparisons to draw solid conclusions.

Since the opportunities to sample biofilms from field distribution systems are limited, most published studies have used model distribution networks and removable coupons over short periods, from days to months [30,36,37], or have used faucets and water meters as alternatives for sampling the biofilms of field DWDSs [38,39]. The limitations of such studies have been clearly described in long-term (three years) studies of model systems [40], field studies of mature biofilms (> 20 years) [34,35], and a simulation study of the influence of hydraulic regimes [21]. However, a study period of three years is still too short to examine mature biofilms in field DWDSs. Further, the field studies of mature biofilms in Germany focused mainly on a small distribution zone within a campus (seven out of eight samples), with the authors attributing the similarity of biofilms on different pipe materials to the fluence of adjacent biofilm communities [34].

In this study, to investigate the long-term influences of pipe materials, planktonic bacteria and mature biofilms (pipe age > 40 years) were sampled from different pipe materials in three distribution areas supplied by the same drinking water treatment plant. The pipe materials included unplasticized polyvinyl chloride (PVC-U), asbestos cement (AC) and grey cast iron (GCI). Our findings offer valuable insights into the long-term influences of pipe materials on biofilms in DWDSs and contribute to our understanding of biofilm development. Moreover, these findings highlight the importance of long-term studies and demonstrate the potentially masked harmonizing process with bacterial community succession over many years.

## 2 Materials and methods

### 2.1 Description of the drinking water supply system

At the Katwijk treatment plant of Dunea, Den Haag, the Netherlands, the source water is transported 30 km to a dune area that contains natural lakes for natural infiltration. After an average residence time of two months, the infiltrated water is extracted and post-treated by softening, powdered activated carbon filtration, aeration, rapid sand filtration, and slow sand filtration. Then, the treated water is pumped into the distribution system. Chlorination and the use of disinfectant residuals are avoided in the Netherlands.

### 2.2 Sampling program

**Fig. 1:**
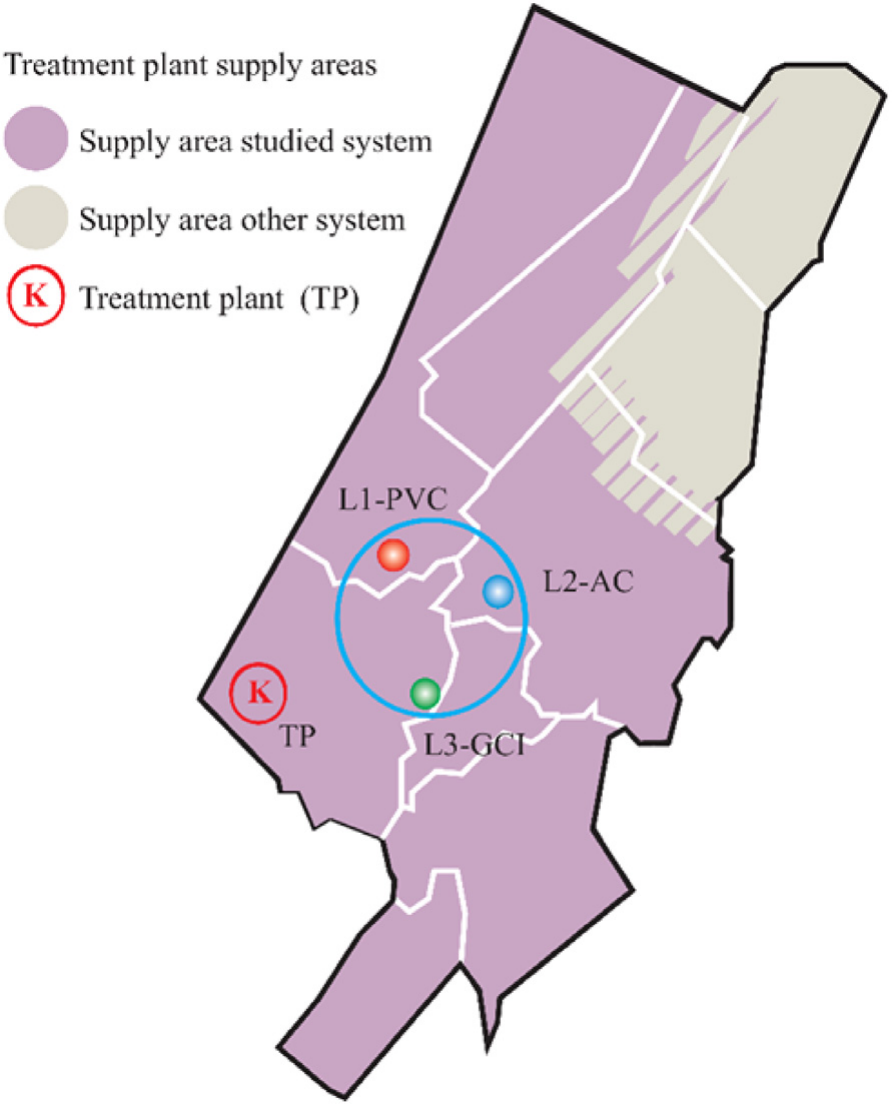
**Layout of the distribution area and sampling locations**. L1, PVC-U pipe (1978, 42 years old); L2, AC pipe (1962, 58 years old); L3, GCI pipe (1970, 50 years old).

As illustrated in Fig. 1, planktonic bacteria were sampled at the treatment plant and three distribution sites (TP, L1, L2 and L3, *n* = 4), while biofilm samples were taken in triplicate from three distribution areas (*n* = 9). The pipe material at L1 was PVC-U, at L2 it was AC, and at L3 it was GCI. The pipe diameter at all three locations was 110 mm and the pipe age was 42 years, 58 years and 50 years, respectively. For planktonic bacteria sampling, 500 mL of water that was stagnant in the pipe for more than three weeks was collected at each sampling point. For biofilm sampling, three sections (length = 30 cm) were cut from the same pipe at each distribution point to sample the biofilm. Two sections were swabbed immediately after pipe cutting, with a swabbing area of approximately 10 cm^2^ positioned at least 5 cm from the cut end to minimize the risk of biofilm disturbance or contamination from the chop saw. One section was sealed with pre-disinfected caps and filled with 1 L of DNA-free water (Millipore) to keep the inner surface wet during transport. All samples were stored at 0 °C and transported to the laboratory within four hours. To detach the bacteria from the biofilm, the pipes were pre-treated by ultrasonication three times for two minutes each time at 42 KHz [41]. The obtained suspensions were used for further DNA extraction and sequencing. Therefore, the triplicate biofilm samples for the same pipe material consisted of duplicate swab-wiped biofilm samples and one ultrasound-obtained biofilm sample.

### 2.3 DNA extraction, illumina sequencing and data processing

The water samples and obtained suspension biofilm samples were filtered through 0.2 μm polycarbonate membrane filters (Whatman, UK). DNA was recovered from the filters or rayon swabs using a FastDNA Spin Kit for Soil (Q-Biogene/MP Biomedicals, Solon, OH, USA), following the manufacturer's instructions [42,43]. The V3-V4 region was amplified with the bacterium-specific forward primer 341F (5′-CCTACGGGNGGCWGCAG-3′) and the reverse primer 805R (5′-GACTACHVGGGTATCTAATCC-3′) [44]. Sequencing was performed on an Illumina Life Sciences GS FLX series genome sequencer (Roche, Switzerland). The obtained DNA sequences were deposited in the DDBJ sequence read archive (Accession Number: PRJNA648471).

The sequences generated from the Illumina Miseq analysis of the 16S rRNA gene amplicons were processed (i.e., filtered, clustered, and taxonomically assigned and aligned) using the Quantitative Insights Into Microbial Ecology (QIIME2, v2018.6) pipeline with the default settings [45,46]. Raw sequences were first processed using DADA2 [47], including quality filtering, denoising, paired-end sequence merging and chimera filtering. DADA2 generated unique amplicon sequence variants that were equivalent to 100% similarity operational taxonomic units (OTUs) in conventional practice. In this publication, we still use the term OTU for the purpose of simplicity (referred to as Feature elsewhere). Taxonomy was assigned using the q2-feature-classifier [48], customized for the primer set used in this study with Silva SSU database release 132 [49]. Multiple sequence alignment and phylogenetic tree construction were performed using the QIIME 2 plugin q2-phylogeny. Alpha and beta diversity analyses were performed using the QIIME 2 plugin q2-diversity.

Weighted and unweighted UniFrac distance matrices were constructed from the phylogenetic tree and used to conduct a principal coordinate analysis (PCoA) [50]. The dominant OTUs were defined as the OTUs with a defined cut-off of relative abundance (> 1%) within each phase/pipe. The significance of beta diversity differences among different sample categories was determined by the PERMANOVA test in QIIME2. Differences were considered statistically significant when the p-value was lower than 0.05 (*p* < 0.05). Venn diagrams exhibiting the similarity of the microbial populations among distinct sample categories were drawn using the VennDiagram package in *R* (3.5.3).

### 2.4 Neutral community model (NCM)

To explore the contributions of neutral processes and environmental selection to the assembly of the filter communities, an evolved NCM following null hypothesis was performed [51]. Specifically, the bulk water samples were considered to be the source community, whereas the biofilm samples were the local target communities. The empirically observed frequency of detection was expressed as the number of biofilm samples in which a target OTU was detected over the total number of biofilm samples. In the implementation of this model, only shared OTUs between the target and source communities were employed. Consequently, the expected frequency of detection in the target communities, which were present via dispersal and ecological drift, was calculated following a beta probability distribution [52]. The neutral model was constructed by 95% binomial confidence intervals based on the Wilson method with the Hmisc package in R [51]. Theoretically, OTUs that fell between the confidence interval were considered to be a result of the neutral dynamics of stochastic births and deaths within the local communities and stochastic immigration from the source communities, according to the neutrality assumption. OTUs falling outside the upper or lower bound of the confidence interval were detected at disproportionately higher or lower frequencies in the local communities than predicted by the neutral model, based on their relative abundance in the source communities, which are advantaged or disadvantaged by the local environment [53].

## 3 Results

In total, 333,660 sequences were generated from the 13 samples (four water and nine biofilm), and these were assigned as 10,431 OTUs. The rarefaction curves reached a plateau after 4000 sequence reads were obtained, indicating that enough sample coverage was obtained in this study (Fig. S1).

### 3.1 Number of observed OTUs

**Fig. 2:**
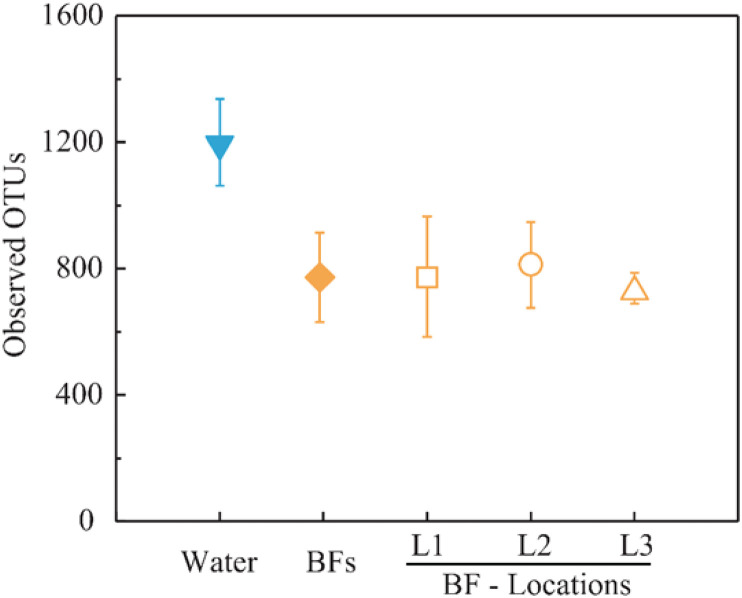
**The number of observed OTUs in water samples from all locations (*****n*****=4) and biofilms sampled from pipes comprising different materials (L1–PVC,*****n*****=3; L2–AC,*****n*****=3; L3–GCI,*****n*****=3)**.

Fig. 2 shows the number of OTUs observed from the water and biofilm samples. On average, 1205 OTUs (*n* = 4) were observed in the water samples, which was much higher than that observed in the biofilm samples (773 OTUs, on average, *n* = 9). For the biofilms formed on different pipe materials at the three locations, 814, 775 and 730 OTUs were observed for AC (L2), PVC-U (L1) and GCI (L3), respectively. The difference in the number of OTUs between water and biofilms was statistically significant, while the differences among the biofilms on different materials were not significant.

### 3.2 Bacterial community composition

At the phylum level, both water and biofilm samples were dominated by Proteobacteria, the relative abundance of which was higher in the biofilms (36.2%−46.1%) than in the water (15.5%–25.2%) (Fig. S2). The community of bacteria in the water samples was dominated by OD1 (16.4%−20.1%), OP3 (3.0%−3.6%), Acidobacteria (1.9%−2.3%), Planctomycetes (1.6%−2.2%), Nitrospirae (0.9%−2.8%), Chlamydiae (1.0%−2.4%), Bacteroidetes (0.8%−2.3%) and TM6 (0.5%−1.5%), in descending order. For the biofilm samples, the bacterial community was dominated by Planctomycetes (5.4%−11.1%), Acidobacteria (3.2%−6.3%), Actinobacteria (1.5%−5.7%), Nitrospirae (2.2%−4.4%), Chloroflexi (2.0%−3.7%), OD1 (0.7%−4.1%) and Gemmatimonadetes (0.9%−3.0%). Importantly, at the phylum level, minor differences were observed among the biofilms on the different pipe materials (PVC-U, AC and GCI).

**Fig. 3:**
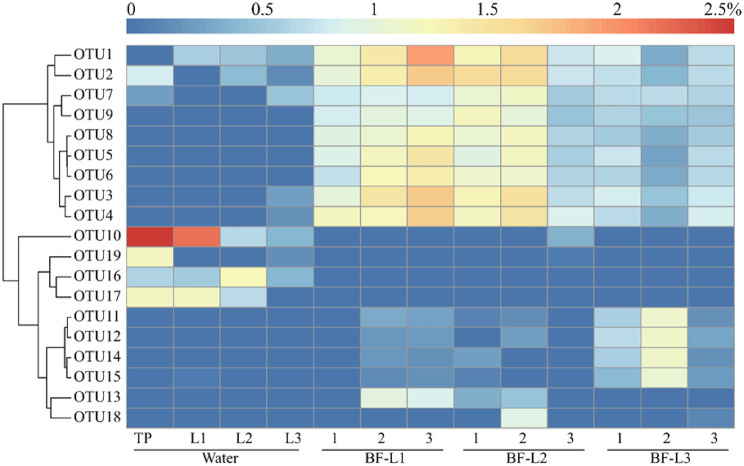
**Heatmap showing the dominant OTUs and their relative abundances in all samples**. The complete list of relative abundances and taxonomy information is provided in Fig. S3.

A total of 19 core OTUs were detected in the water and biofilm samples (Fig. 3; Table S2). In the water samples, OTU10 (f_Hyphomicrobiaceae) and OTU16 (f_Hyphomicrobiaceae) were the most dominant OTUs (relative abundance 0.5%−2.7%; occupancy, 100%). OTU16 was only detected in the water samples and not in any of the biofilm samples. For the biofilm samples across all locations and pipe materials, the core OTUs included OTU1, OTU2, OTU3, OTU4, OTU5, OTU6 and OTU8 which were assigned to the class Gammaproteobacteria (relative abundance, 0.4%−2.1%; occupancy, 100%), and OTU7 and OTU9, which were assigned to *Nirospira* spp. (relative abundance, 0.6%−1.2%; occupancy, 100%). OTU5, OTU6, OTU8 and OTU9 were detected only in biofilm samples, not in water samples.

When comparing the dominant OTUs among the biofilms formed on different pipe materials, it can be seen that most of the dominant OTUs were shared by all pipe materials (13/17 OTUs, Venn gram Fig. S4). However, two OTUs (OTU10, OTU19) were detected only in biofilms formed on AC pipe; they were not detected in biofilms on the PVC-U and GCI pipes. Similar to the observations at the phylum level, there were minor differences in the dominant OTUs of the biofilms formed on PVC-U, AC and GCI pipes, indicating minor effects of the pipe material on biofilm formation.

### 3.3 Bacterial community similarity

**Fig. 4:**
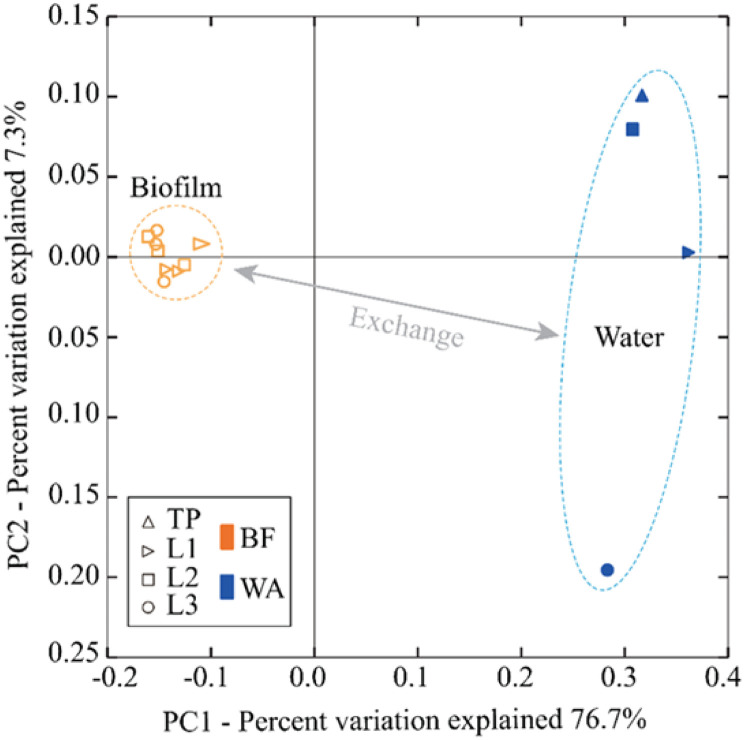
**PCoA plot generated using the unweighted UniFrac distance matrix showing the microbial community distributions of different sample categories**.

The PCoA plot based on unweighted UniFrac distances clearly shows the two clusters of water and biofilm (Fig. 4; Table 1, *p* < 0.05). The bacterial communities of the water samples were clearly distanced from each other, suggesting clear variation in bulk water bacteria among the sampling locations. Moreover, it can be seen that the biofilm samples were clustered closely together, highlighting the high reproducibility of the obtained results (the triplicate samples from each pipe material) and the weak influence of the pipe material on the bacterial communities of biofilms. This is consistent with the above findings on the composition of the bacterial communities.

**Table 1:** **Influences of the pipe material tested by two-way ANOSIM**.

	Group 1	Group 2	*p*-value
Tests	BF	WA	0.04
	L1 L1	L2	0.39
		L3	0.10
	L2	L3	0.10

The beta diversity results for the biofilm samples from different locations and different sampling strategies are presented in a PCoA plot (Fig. S3). The bacterial communities of the biofilm samples exhibited little variation between the different locations, with the PCoA plot explaining very little of the variation. From the Venn diagram (Fig. S4), it can be seen that there were differences in OTU10, OTU13, OTU18 and OTU19. A comparison of biofilm samples collected by pipe specimen ultrasound versus swab indicates that there was variation between the different sampling strategies in the same section (*p* < 0.01, PERMANOVA test by QIIME2). The biofilm swab samples were clustered together in different locations, demonstrating the reproducibility of swab samples.

### 3.4 Effects of neutral processes on biofilm microbial community assembly

**Fig. 5:**
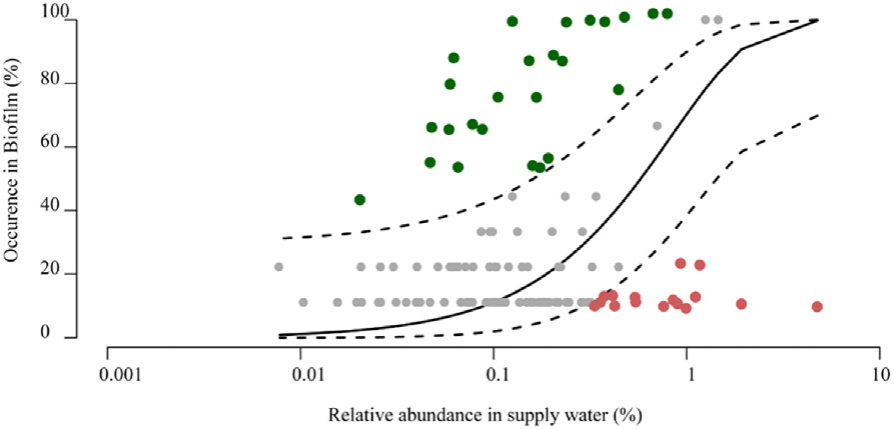
**Neutral community model for the combined biofilm samples (*n* = 9)**. The solid line is the model prediction, and the dashed lines represent the 95% confidence intervals. The green points represent the OTUs for which the observed frequency is greater than the model prediction (enriched), and the red points represent the OTUs for which the observed frequency is less than the prediction (disadvantaged), based on their mean relative abundances in the supply water communities.

Between the 4694 OTUs and 5879 OTUs detected in water (over a three-week-period) and biofilms (> 40 years old), there were only 142 shared OTUs that accounted for 2.4% of the number of observed OTUs and 13.2% of the total sequences in the biofilms. To further explore the microbial community assembly within the biofilms, those 142 shared OTUs were used to calculate the probability of detecting the OTUs in the biofilm due to neutral processes, e.g., dispersal and ecological drift (Table 2; Fig. 5). Though the number of neutral process-driven OTUs accounted for 71.1% of the total number of shared OTUs, they accounted for just 35.2% of the shared sequences. Further, 28.9% of the number of shared OTUs that were environmentally selected accounted for 62.6% of the shared sequences, including OTU3, OTU4, OTU7, OTU10 and OTU11; these had relative abundances > 1% and occupancies of 100%. Moreover, the goodness-of-fit (*R*^2^) value was 0.02 (where ≤ 0 is no fit and 1 is a perfect fit). This further confirms that taking the water microbes as a meta-community, the assembly of the biofilm bacterial community is governed by environmental selection rather than neutral processes.

**Table 2:** **Spearman rank correlation coefficients**.

	OTUs	Sequences	Relative Abundance	
Water	4694	73,527	11.5% (unique)	
Biofilms	5879	260,133	86.8% (unique)	
Shared	142	34,248	13.2% (biofilm)	
Neutral	101	12,069	4.6% (biofilm)	35.2% (share)
Enriched	25	21,443	8.3% (biofilm)	62.6% (share)
Disadvantaged	16	736	0.3% (biofilm)	2.2% (share)

## 4 Discussion

Different from the traditionally used pilot/simulated systems and/or young biofilm sampling, this study investigated mature biofilms on full-scale drinking water distribution pipes comprised of different materials (i.e., PVC-U, AC and GCI) that were installed in different areas supplied by the same water treatment plant. Here, the long-term effects are discussed in terms of the influences of pipe material on biofilm formation in drinking water pipes, and in particular, the possible harmonizing effects on the bacterial community assembly.

### 4.1 Microbiome assembly in drinking water biofilms revealed by the NCM model

The NCM model results based on the shared OTUs between water and biofilms suggested that the assembly of the biofilm bacterial community was governed by environmental selection, while water microbes comprise a meta-community that serves as a seed bank. This verifies the hypothesis proposed by Henne et al. [34]. More specifically, the enriched community member OTU7, assigned as *Nitrospira* spp., has been widely reported to be able to thrive in drinking water biofilms [34,40,50]. In contrast, disadvantaged community members, such as OTU14 and OTU40, assigned to the family Hyphomicrobiaceae, have been found to thrive in phosphorus-limited environments and form filamentous biofilms in drinking water biofilters [54]. They are disadvantaged because the Dutch drinking water supply system is carbon-limited, with the pursuit of a chlorine-free drinking water supply and extremely low assimilable organic carbon (AOC < 10 μg C/L) [55].

However, it should be mentioned that the number of shared OTUs between the water and biofilm samples (142 OTUs) was low in the present study. This is because of the low number of water samples. The biofilms sampled in this study had developed over four decades under historical water microbiology conditions. Since historical water samples were not available and the acquired water samples were only snapshots, higher temporal resolution could not be achieved for this study. Moreover, there was significant variation among the bacterial communities in the bulk water samples, which might be caused by stagnation and the contributions of plumbing systems [[56], [57], [58]]. Since the shared OTUs between the water and biofilm samples were selected for the NCM analysis, the model results would not be influenced. For future research, the combination of dynamic water and biofilm sampling at a certain frequency over a long period of time would offer more valuable insights into microbiome assembly over time. In particular, feeding such a high-resolution data set into the NCM model would assist in uncovering the essential mechanism underlying biofilm formation and strategies for its control [53,56].

### 4.2 Influence of pipe material on biofilm formation

As revealed by the composition (Fig. 3) and diversity of the bacterial communities (Fig. 4), though there were slight differences in terms of certain members and their presence and abundance, the mature biofilm communities, which were more than 40 years old, formed on different pipe materials (i.e., PVC-U, AC and GCI) were highly similar. This suggests that the pipe material has only a minor influence on the bacterial communities of biofilms. However, there is a general consensus among the academic community that the pipe material is important for both the quantity and community of biofilms [31,36,59]. The lack of differences as a function of pipe material in the present study are in contrast to the commonly observed influences of the pipe material on both the composition and diversity of biofilm bacterial communities in water supply pipes, such as plumbing systems (28-day-old biofilm) [60], shower hoses (eight-month-old biofilm) [31], and modelled and field distribution systems (biofilms ranging in age from 1 month to 42 weeks) [29,32,61]. However, the above studies all investigated young biofilms that were less than one year of age.

On the other hand, studies that have reported a similar bacterial quantity and community in mature biofilms formed on different pipe materials were conducted in an office building in Finland (copper vs. PEX, biofilms greater than one year old) [33] and in a main distribution pipe in Germany (steel, copper, PVC, biofilms more than 20 years old) [34]. Both of the above studies illustrate the potential importance of the vicinity of the biofilm over the support material. Placing different coupon materials in the same reactor, Aggarwal et al. also found that the coupon material did not have a significant impact on the biomass level or composition of the biofilm community [62]. By comparing their results with a similar study that used separate reactors for each coupon material [63], the authors argued that isolating different materials to study their impacts on biofilms cannot mimic full-scale systems containing a variety of materials [62], as isolation of a material neglects to consider the mutual influences of biofilms via the exchange of bacteria through mitigation and/or diffusion [34,64].

In the present study, the biofilms on three pipe materials were taken from different supply areas that were > 10 km away from each other. Our findings are consistent with an earlier study of the full-scale chloraminated DWDS of Saint Paul, Minn, USA. This study observed surprisingly similar biofilm communities regardless of the age, location and pipe material (unlined cast iron versus cement-lined cast iron, > 53-year-old biofilm) [35]. It is interesting that the two studies both observed similar bacterial communities on different pipe materials from different locations (spatially distanced), though the present study of the Dutch system was completely different to the system in Saint Paul, especially in relation to the disinfection strategies (unchlorinated vs. chloraminated). The key common factor is that both studies investigated mature biofilms greater than 40 years of age. It is rational to hypothesize that years-long (decades-long) acclimatization harmonized the initial significant differences induced by the pipe material. This hypothesis is supported by the previous observation of less-pronounced differences in terms of the bacterial quantity and community of biofilms formed on four out of six materials after eight months [31]. This may be explained by a reduction in the nutrients leaching from the pipe and, subsequently, biofilm formation governed by the microbes and nutrients in the supply water. Similarly, the microbial communities of pipe biofilms from different water sources were different, whereas those of different pipe materials from the same water source were similar in a Dutch chlorine-free DWDS [65]. In contrast, plastic pipe biofilms from kitchens and bathrooms have been found to have different microbial communities due to the different operational conditions (e.g., water physical chemistry, hydraulic condition) of the supply water [66,67].

### 4.3 Practical implications

To ensure biosafety, considerable attention and effort have been invested in understanding the formation of biofilms and in the application of biofilm management strategies for drinking water distribution networks over decades. Pipe material has been considered to be a key factor possibly governing the potential for biofilm formation and bacterial community assembly [27]. However, until now, the critical questions of how and how long the pipe material influences biofilm development have remained unanswered. To date, tests evaluating the potential for pipe materials to promote microbial growth have varied from 2 weeks to 16 weeks [28]. In addition, as mentioned above, simulation studies of the influence of pipe materials on biofilm communities have been conducted over periods from days to years. Such big variations in the scale of study time might be the reason for the conflicting observations and conclusions across studies, as well as the reported differences between simulated reactors and full-scale systems that have operated for decades [62]. Therefore, the choice of study time may mask the mechanism by which the pipe material influences biofilm development and bacterial community succession.

As demonstrated in the present study of an unchlorinated Dutch system (> 40-year-old biofilm), a chlorinated German system (> 20-year-old biofilm) [34] and a chlormainated system (> 53-year-old biofilm) [35], biofilm harmonization occurs regardless of the pipe material and other environmental circumstances in full-scale distribution systems, as long as the different pipe materials are supplied with same drinking water. Once the harmonized stable microbial ecology is established, there are potential risks associated with transition effects when the quality of the supply water changes, which may lead to destabilization of the biofilm matrix and sudden release of opportunistic pathogens [[8], [9], [10]].

In addition, from both scientific and practical perspectives, an essential question to be answered is how long the harmonizing process takes before a quantity- and community-stabilized biofilm can be established. Martiny et al. suggested that biofilm formation may take 200–300 days to reach a stationary density and ∼500 days to establish a stable population on stainless steel [40]. Proctor et al. observed less pronounced differences after eight months than in the early months when examining biofilms formed on four of six of the tested flexible polymeric pipe materials, suggesting that the harmonizing time differs for different materials [31]. To determine the time threshold, the long-term efficacy of pipe materials, and other essential drinking water biofilm-related questions, long-term (years-long) studies of the dynamics of biofilm formation using the latest developed high-throughput quantification and sequencing techniques together with high-resolution water-biofilm paired sampling and microbial ecology models are required.

## 5 Conclusion

As demonstrated by the number of observed OTUs, the bacterial community in the bulk water was more diverse than that of the biofilms. The mature biofilm bacterial communities on PVC-U, AC and GCI pipes were highly similar in terms of the alpha and beta diversity, indicating a minor influence of the pipe material on the biofilm. As revealed by the NCM model, biofilm community assembly was driven by environmental selection rather than a neutral process. Members of *Nitrospira* spp. were enriched, while members of the family Hyphomicrobiaceae were disadvantaged. The long-term effects of the pipe material on biofilm formation and the harmonizing process require further exploration.

## Declaration of competing interest

The authors declare that they have no conflicts of interest in this work.

## Appendix Supplementary materials

Image, image 1 dummy alt text

Image, image 2 dummy alt text

Image, image 3 dummy alt text

Image, image 4 dummy alt text

Image, image 5 dummy alt text

Image, image 6 dummy alt text

Image, image 7 dummy alt text

Image, application 8
