# Supplementary figures and images for: Long-term influences of pipe materials on bacterial communities of matured biofilms (> 40 years’ old) in drinking water distribution systems
Source: Fundam Res. 2024 Jun 29;6(4):2369–75. doi: 10.1016/j.fmre.2024.05.019 (PMC13424401; doi:10.1016/j.fmre.2024.05.019)

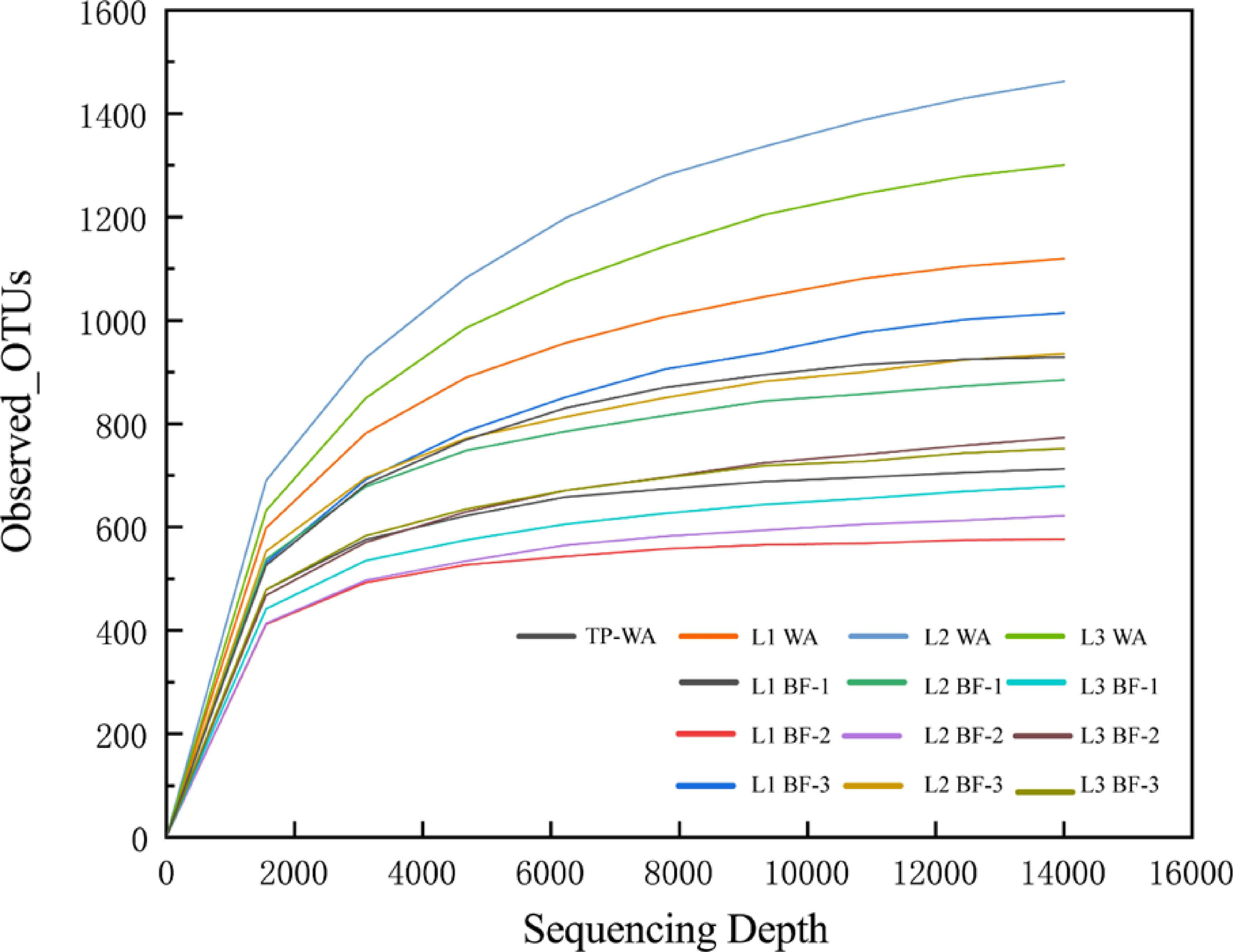

Supplement: Supplementary file 1 [file mmc1.jpg]

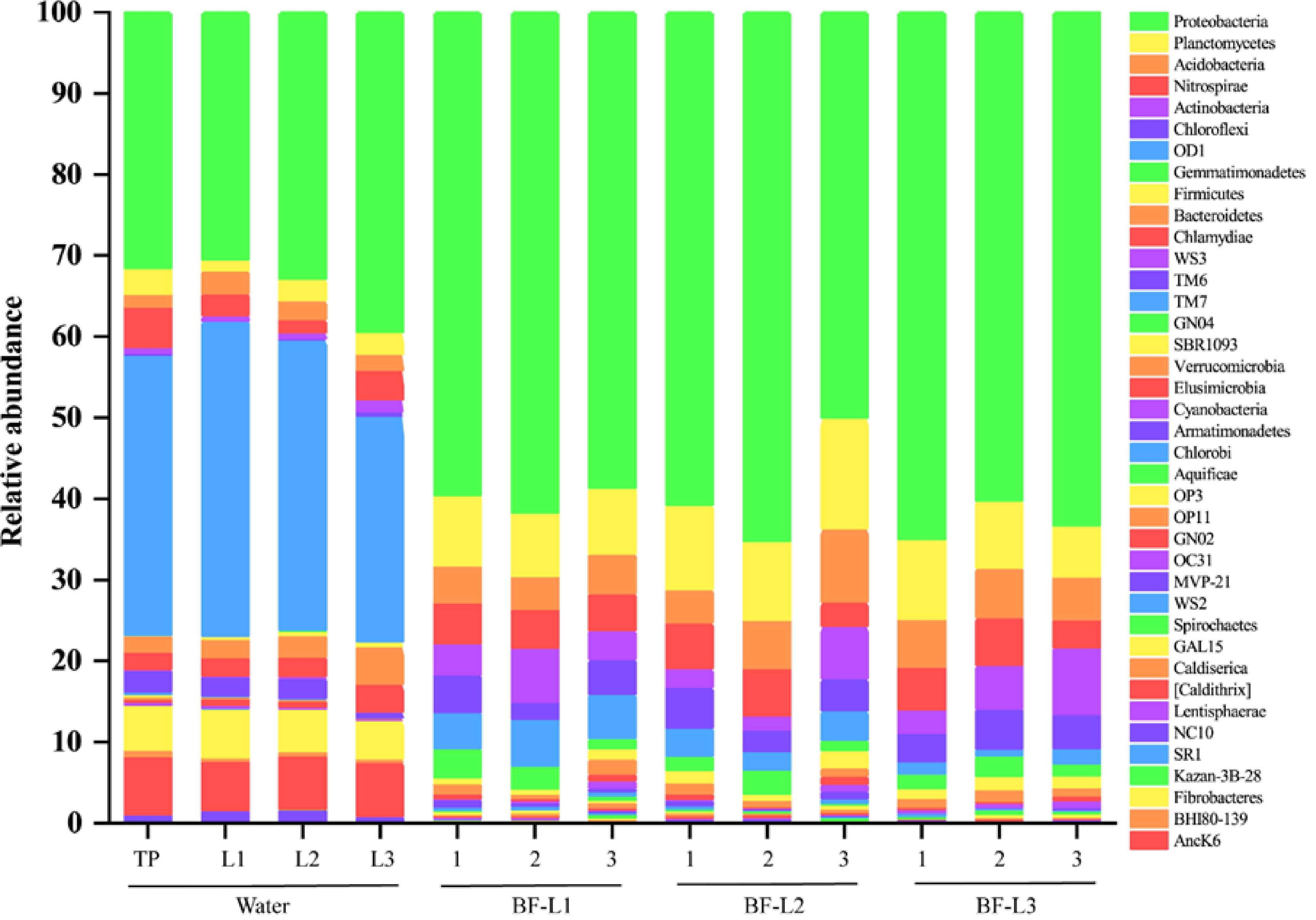

Supplement: Supplementary file 2 [file mmc2.jpg]

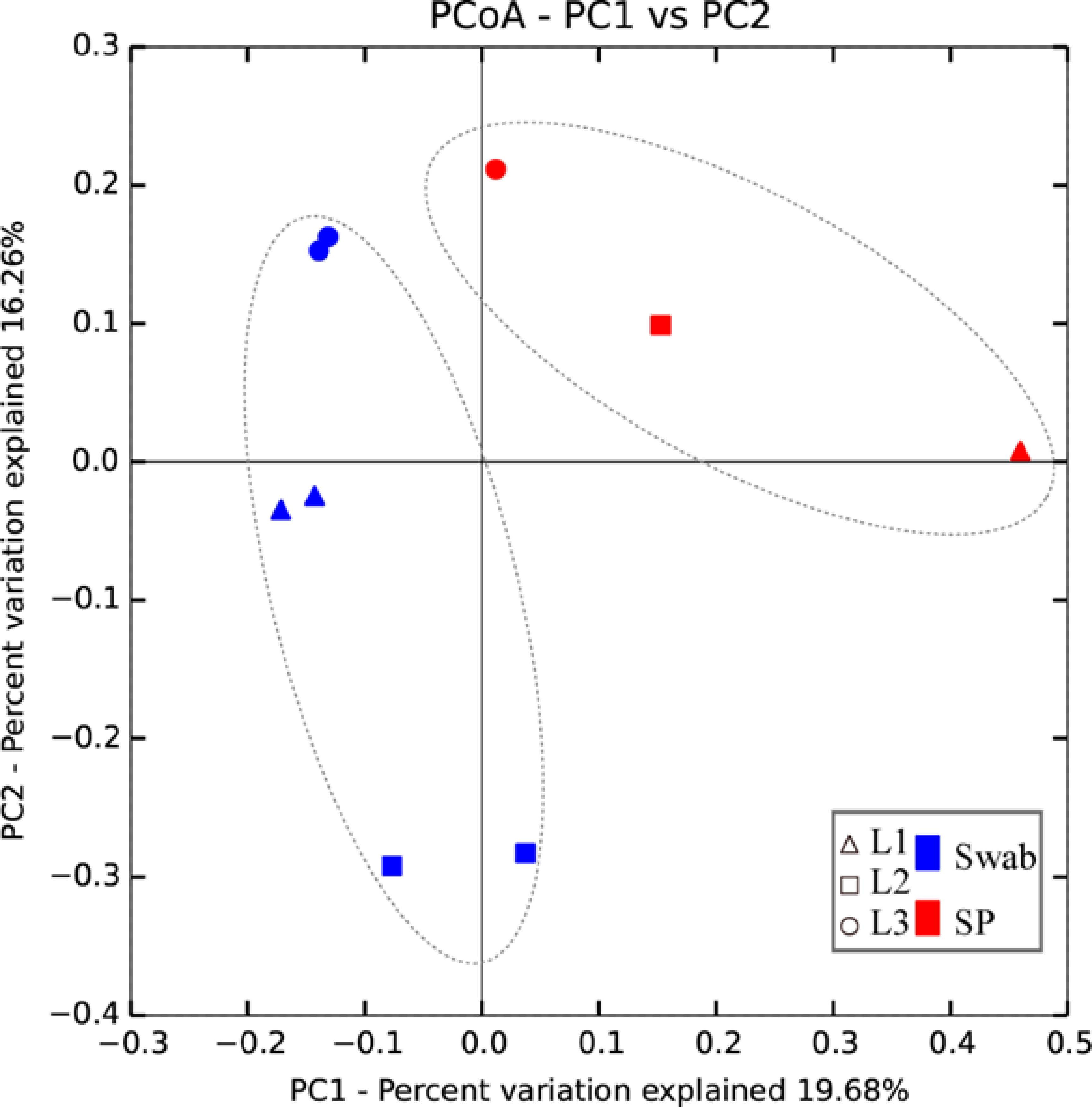

Supplement: Supplementary file 3 [file mmc3.jpg]

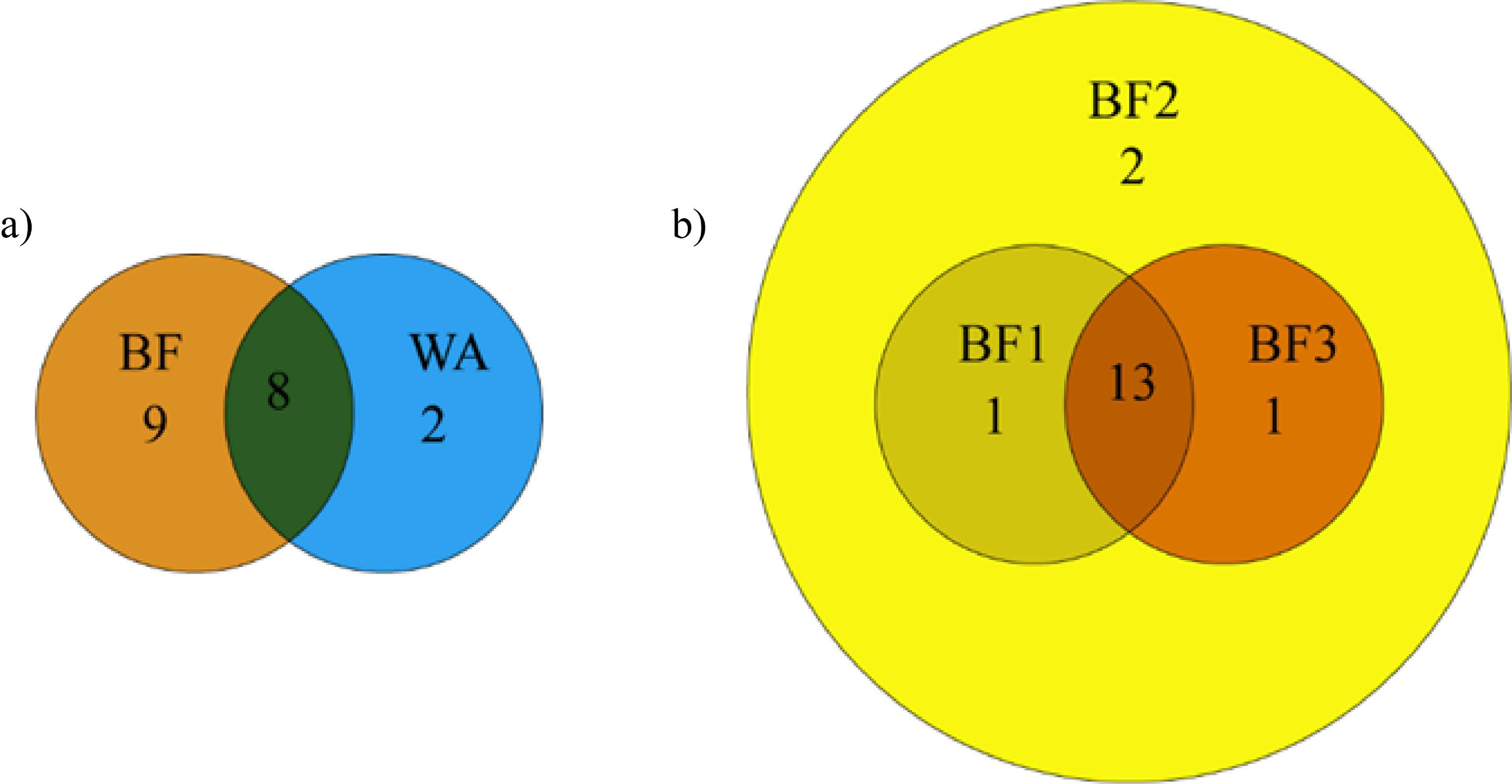

Supplement: Supplementary file 4 [file mmc4.jpg]

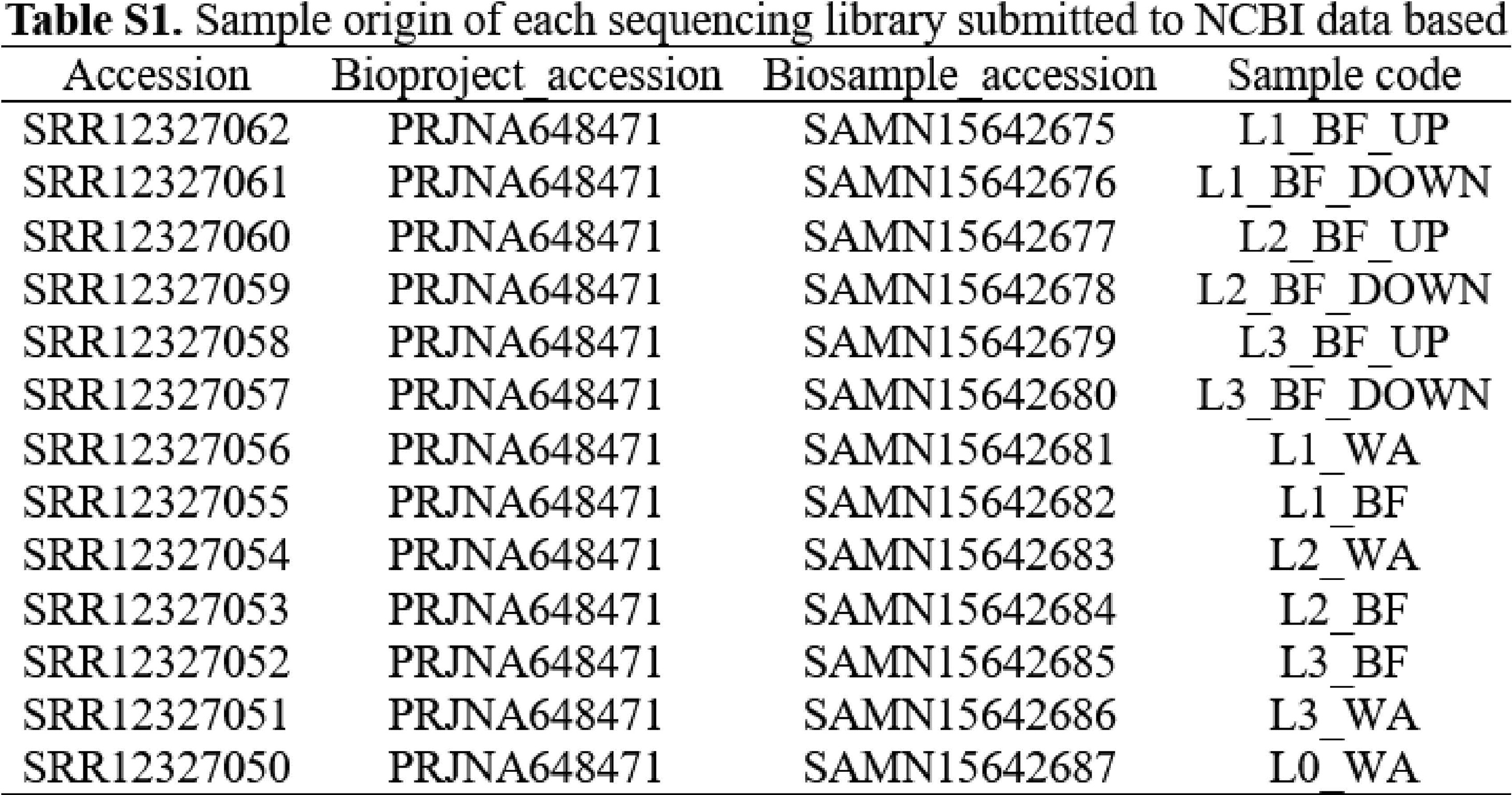

Supplement: Supplementary file 5 [file mmc5.jpg]

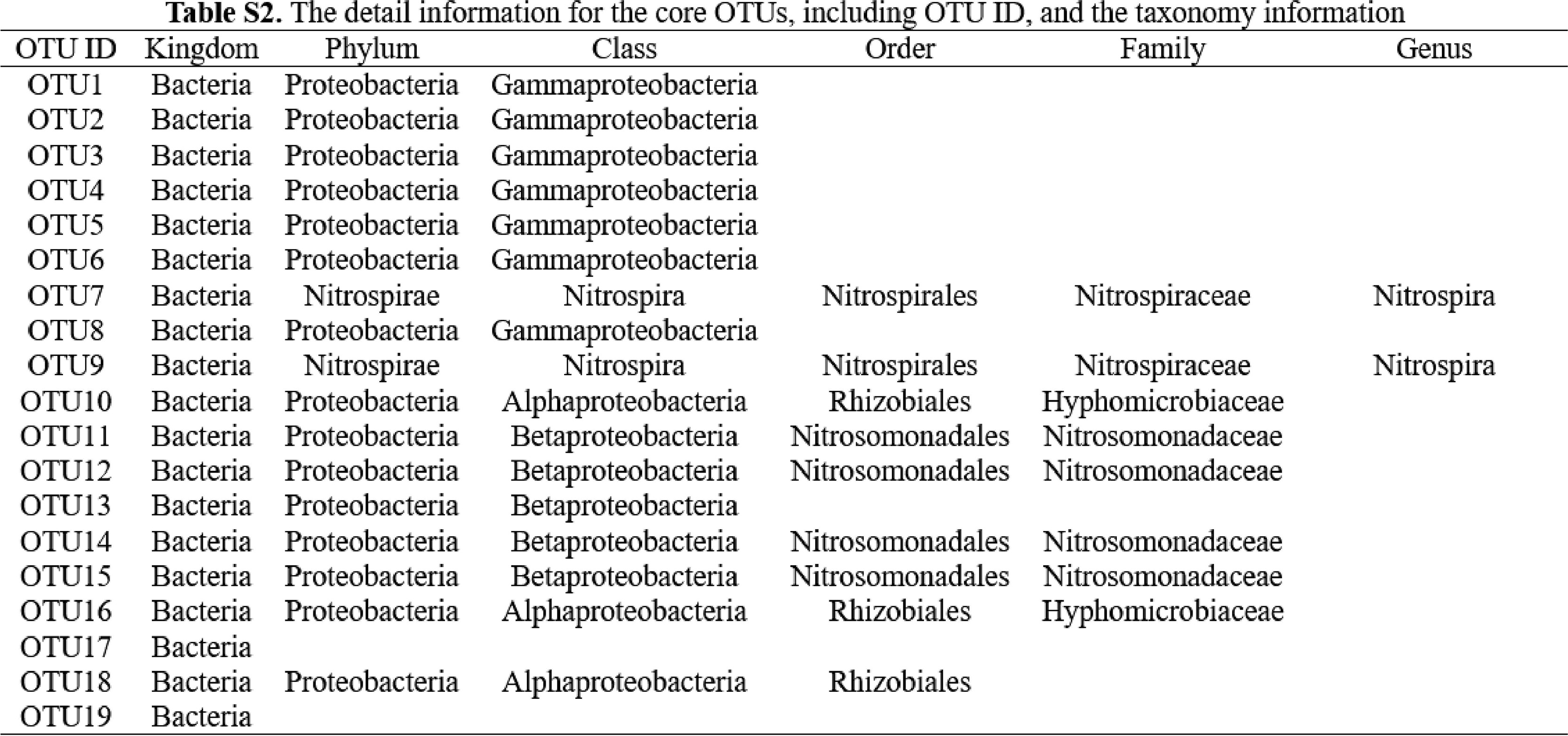

Supplement: Supplementary file 6 [file mmc6.jpg]

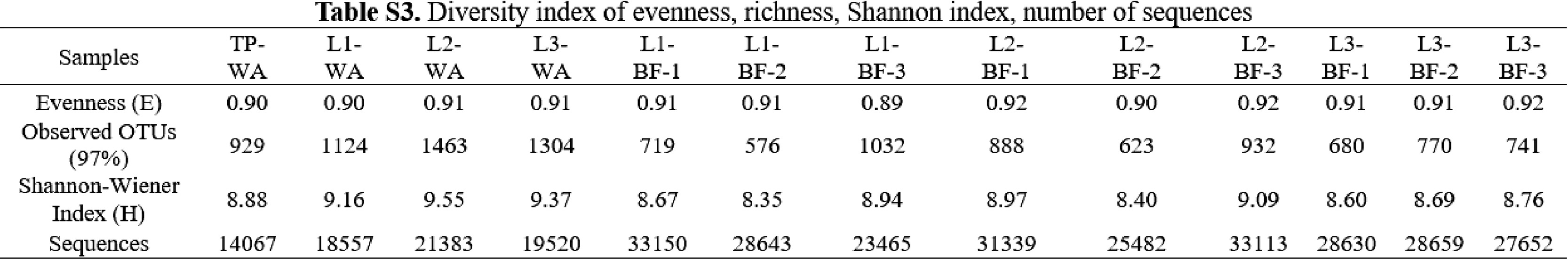

Supplement: Supplementary file 7 [file mmc7.jpg]
