# Supplementary material for: Long-term influences of pipe materials on bacterial communities of matured biofilms (> 40 years’ old) in drinking water distribution systems
Source: Fundam Res. 2024 Jun 29;6(4):2369–75. doi: 10.1016/j.fmre.2024.05.019 (PMC13424401; doi:10.1016/j.fmre.2024.05.019)
Supplement: Supplementary file 8 [file mmc8.docx]

Supplementary Information for:

**Long-term influences of pipe material on bacterial communities of matured biofilms (> 40 years’ old) in drinking water distribution systems**

Anran Ren ^a,b^, Jun Li ^a,b^, Zhen Zhang ^a,c^, Ed van der Mark ^d^, Lihua Chen ^a,e^, Xiaoming Li ^a,b,*^, Walter van der Meer ^f,g^, Gang Liu ^a,b,e,*^

^a^ Key Laboratory of Drinking Water Science and Technology, Research Center for Eco-Environmental Sciences, Chinese Academy of Sciences, Beijing 100085, China

^b^ University of Chinese Academy of Sciences, Beijing 100049, China

^c^ General Office, the People’s Government of Xinglong prefecture, Chengde 067300, China

^d^ Dunea Water Company, Zoetermeer P.O. Box 756, the Netherlands

^e^ Sanitary engineering, Department of Water management, Faculty of Civil Engineering and Geosciences, Delft University of Technology, Delft P.O. Box 5048 , the Netherlands

^f^ Science and Technology, University of Twente, Enschede P.O. Box 217, the Netherlands

^g^ Oasen Water Company, Gouda P.O. Box 122, the Netherlands

***Corresponding authors.**

E-mail address: xmli@rcees.ac.cn (X.M. Li), gliu@rcees.ac.cn (G. Liu).

The supplementary information includes 3 tables and 4 figures in 8 pages.

**Table S1.** Sample origin of each sequencing library submitted to NCBI data based

| Accession | Bioproject_accession | Biosample_accession | Sample code |
| --- | --- | --- | --- |
| SRR12327062 | PRJNA648471 | SAMN15642675 | L1_BF_UP |
| SRR12327061 | PRJNA648471 | SAMN15642676 | L1_BF_DOWN |
| SRR12327060 | PRJNA648471 | SAMN15642677 | L2_BF_UP |
| SRR12327059 | PRJNA648471 | SAMN15642678 | L2_BF_DOWN |
| SRR12327058 | PRJNA648471 | SAMN15642679 | L3_BF_UP |
| SRR12327057 | PRJNA648471 | SAMN15642680 | L3_BF_DOWN |
| SRR12327056 | PRJNA648471 | SAMN15642681 | L1_WA |
| SRR12327055 | PRJNA648471 | SAMN15642682 | L1_BF |
| SRR12327054 | PRJNA648471 | SAMN15642683 | L2_WA |
| SRR12327053 | PRJNA648471 | SAMN15642684 | L2_BF |
| SRR12327052 | PRJNA648471 | SAMN15642685 | L3_BF |
| SRR12327051 | PRJNA648471 | SAMN15642686 | L3_WA |
| SRR12327050 | PRJNA648471 | SAMN15642687 | L0_WA |

**Table S2.** The detail information for the core OTUs, including OTU ID, and the taxonomy information

| OTU ID | Kingdom | Phylum | Class | Order | Family | Genus |
| --- | --- | --- | --- | --- | --- | --- |
| OTU1 | Bacteria | Proteobacteria | Gammaproteobacteria |  |  |  |
| OTU2 | Bacteria | Proteobacteria | Gammaproteobacteria |  |  |  |
| OTU3 | Bacteria | Proteobacteria | Gammaproteobacteria |  |  |  |
| OTU4 | Bacteria | Proteobacteria | Gammaproteobacteria |  |  |  |
| OTU5 | Bacteria | Proteobacteria | Gammaproteobacteria |  |  |  |
| OTU6 | Bacteria | Proteobacteria | Gammaproteobacteria |  |  |  |
| OTU7 | Bacteria | Nitrospirae | Nitrospira | Nitrospirales | Nitrospiraceae | Nitrospira |
| OTU8 | Bacteria | Proteobacteria | Gammaproteobacteria |  |  |  |
| OTU9 | Bacteria | Nitrospirae | Nitrospira | Nitrospirales | Nitrospiraceae | Nitrospira |
| OTU10 | Bacteria | Proteobacteria | Alphaproteobacteria | Rhizobiales | Hyphomicrobiaceae |  |
| OTU11 | Bacteria | Proteobacteria | Betaproteobacteria | Nitrosomonadales | Nitrosomonadaceae |  |
| OTU12 | Bacteria | Proteobacteria | Betaproteobacteria | Nitrosomonadales | Nitrosomonadaceae |  |
| OTU13 | Bacteria | Proteobacteria | Betaproteobacteria |  |  |  |
| OTU14 | Bacteria | Proteobacteria | Betaproteobacteria | Nitrosomonadales | Nitrosomonadaceae |  |
| OTU15 | Bacteria | Proteobacteria | Betaproteobacteria | Nitrosomonadales | Nitrosomonadaceae |  |
| OTU16 | Bacteria | Proteobacteria | Alphaproteobacteria | Rhizobiales | Hyphomicrobiaceae |  |
| OTU17 | Bacteria |  |  |  |  |  |
| OTU18 | Bacteria | Proteobacteria | Alphaproteobacteria | Rhizobiales |  |  |
| OTU19 | Bacteria |  |  |  |  |  |

**Table S3.** Diversity index of evenness, richness, Shannon index, number of sequences

| Samples | TP-WA | L1-WA | L2-  WA | L3-  WA | L1-  BF-1 | L1-  BF-2 | L1-  BF-3 | L2-  BF-1 | L2-  BF-2 | L2-  BF-3 | L3-  BF-1 | L3-  BF-2 | L3-BF-3 |
| --- | --- | --- | --- | --- | --- | --- | --- | --- | --- | --- | --- | --- | --- |
| Evenness (E) | 0.90 | 0.90 | 0.91 | 0.91 | 0.91 | 0.91 | 0.89 | 0.92 | 0.90 | 0.92 | 0.91 | 0.91 | 0.92 |
| Observed OTUs (97%) | 929 | 1124 | 1463 | 1304 | 719 | 576 | 1032 | 888 | 623 | 932 | 680 | 770 | 741 |
| Shannon-Wiener Index (H) | 8.88 | 9.16 | 9.55 | 9.37 | 8.67 | 8.35 | 8.94 | 8.97 | 8.40 | 9.09 | 8.60 | 8.69 | 8.76 |
| Sequences | 14067 | 18557 | 21383 | 19520 | 33150 | 28643 | 23465 | 31339 | 25482 | 33113 | 28630 | 28659 | 27652 |


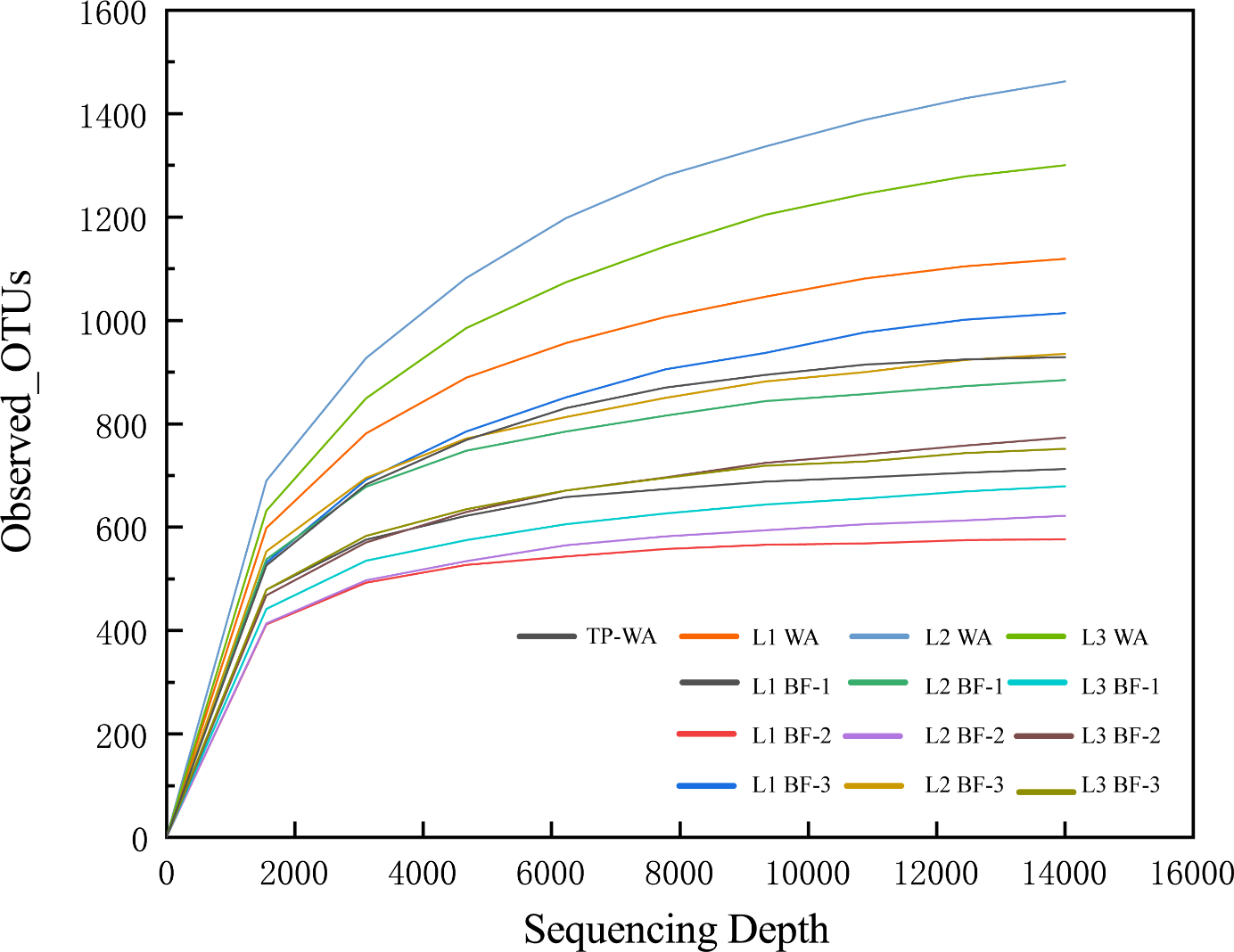
**Figure S1.** *Rarefaction curve of 16s sequences generated by Illumina platform on 13 samples of water and biofilm. In the figure, TP-WA for station water, L1 for sampling location 1 and the pipe material is PVC, L2 for sampling location 2 and the pipe material is asbestos cement, L3 for sampling location 3 and the pipe material is gray cast iron. BF-1 and BF-2 for samples collected by swabbing, BF-3 for sample collected by pipe specimen sonication.*


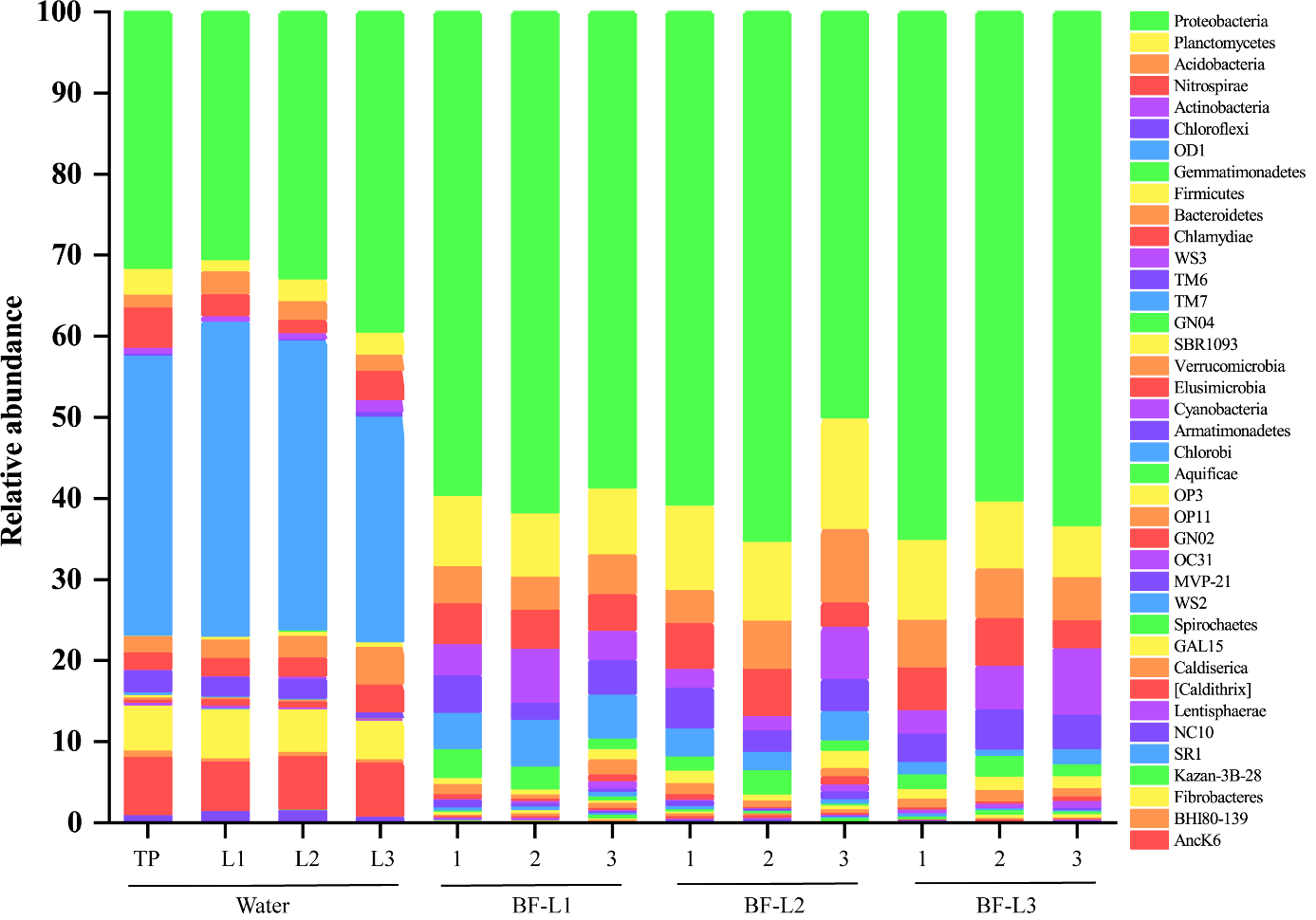


**Figure S2.** *Phylum level composition of water and biofilm bacterial community.*


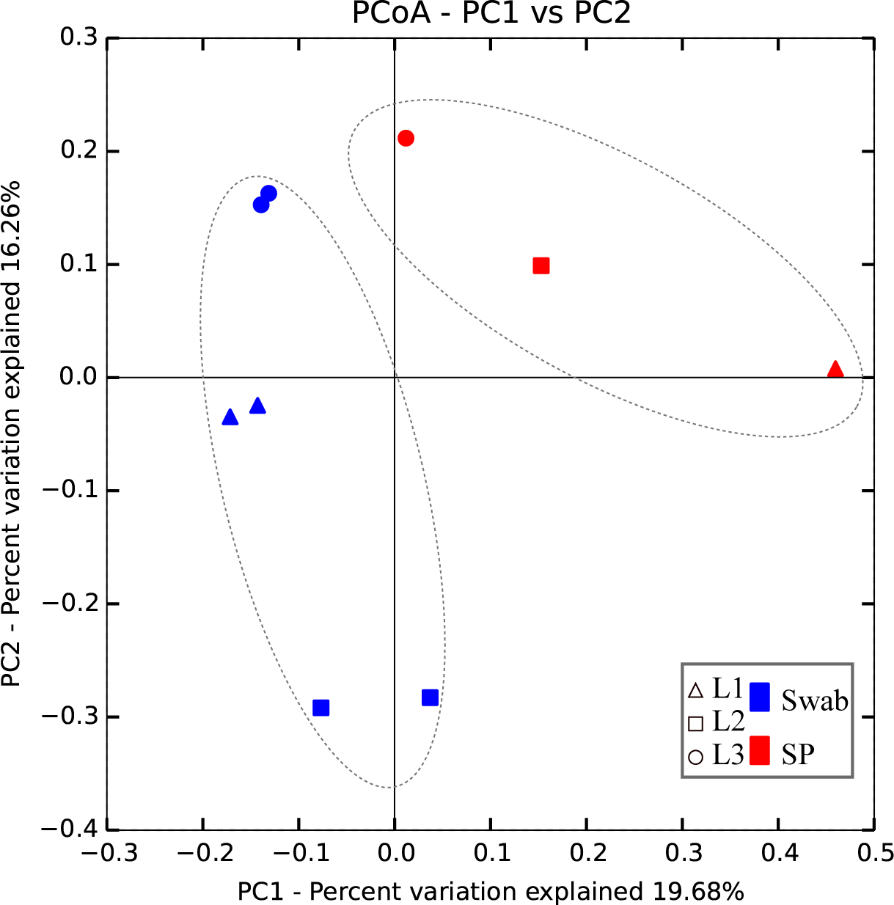


**Figure S3.** *PCoA plot generated using unweighted UniFrac distance matrix showing the biofilm microbial community distribution of different locations and sampling strategies.*

a)
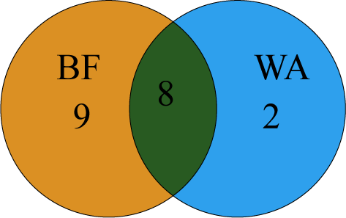
 b)
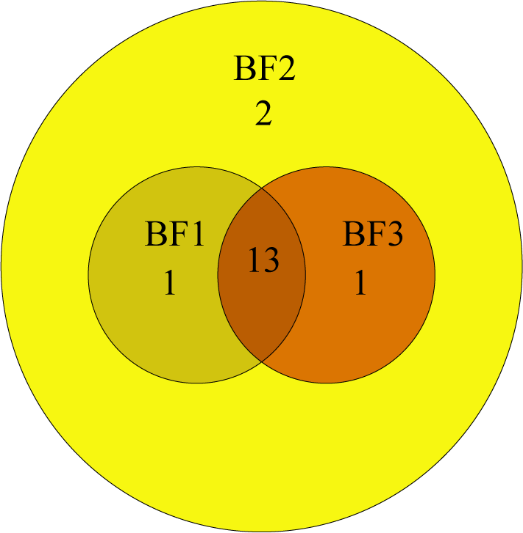


**Figure S4.** *Venn diagram of core OTUs (>1%) associated with water and biofilm in in the four locations: a) sharing of the core OTUs between biofilm and water; b) sharing of the core OTUs between biofilm from three location L1, L2 and L3.*

**a) OTUs shared between water and biofilm:**

9 elements included exclusively in "BF":

OTU5; OTU6; OTU8; OTU9; OTU11; OTU12; OTU13; OTU14; OTU18

2 elements included exclusively in "WA":

OTU16; OTU17

8 common elements in "BF" and "WA":

OTU1; OTU2; TOU3; OTU4; OTU7; OTU10; OTU15; OTU19

**b) OTUs shared between biofilm from location 1, location 2 and location 3.**

1 element included exclusively in "BF1" and “BF2”, not in “BF3”:

OTU13

2 elements included exclusively in "BF2":

OTU10; OTU19

1 element included exclusively in "BF2" and “BF3”, not in “BF1”:

OTU18

13 common elements in "BF1", “BF2”, and "BF3":

OTU1; OTU2; TOU3; OTU4; OTU5; OTU6; OTU7; OTU8; OTU9; OTU11; OTU12; OTU13; OTU14
